# Supplementary material for: Structure of the complete Saccharomyces cerevisiae Rpd3S-nucleosome complex
Source: Nat Commun. 2023 Dec 8;14:8128. doi: 10.1038/s41467-023-43968-8 (PMC10709384; doi:10.1038/s41467-023-43968-8)
Supplement: Supplementary file 1 — Supplementary Information [file 41467_2023_43968_MOESM1_ESM.pdf]

**Supplementary Information For**

# Structure of the complete *Saccharomyces cerevisiae* Rpd3S-nucleosome complex

Jonathan W. Markert[1], Seychelle M. Vos[2*], Lucas Farnung [1*]

[1]Department of Cell Biology, Blavatnik Institute, Harvard Medical School, Boston, MA, USA

[2]Department of Biology, Massachusetts Institute of Technology, Cambridge, MA, USA

[*]Co-corresponding authors. Correspondence should be addressed to S.M.V (seyvos@mit.edu) and L.F. (Lucas_Farnung@hms.harvard.edu). Requests for materials should be addressed to L.F.

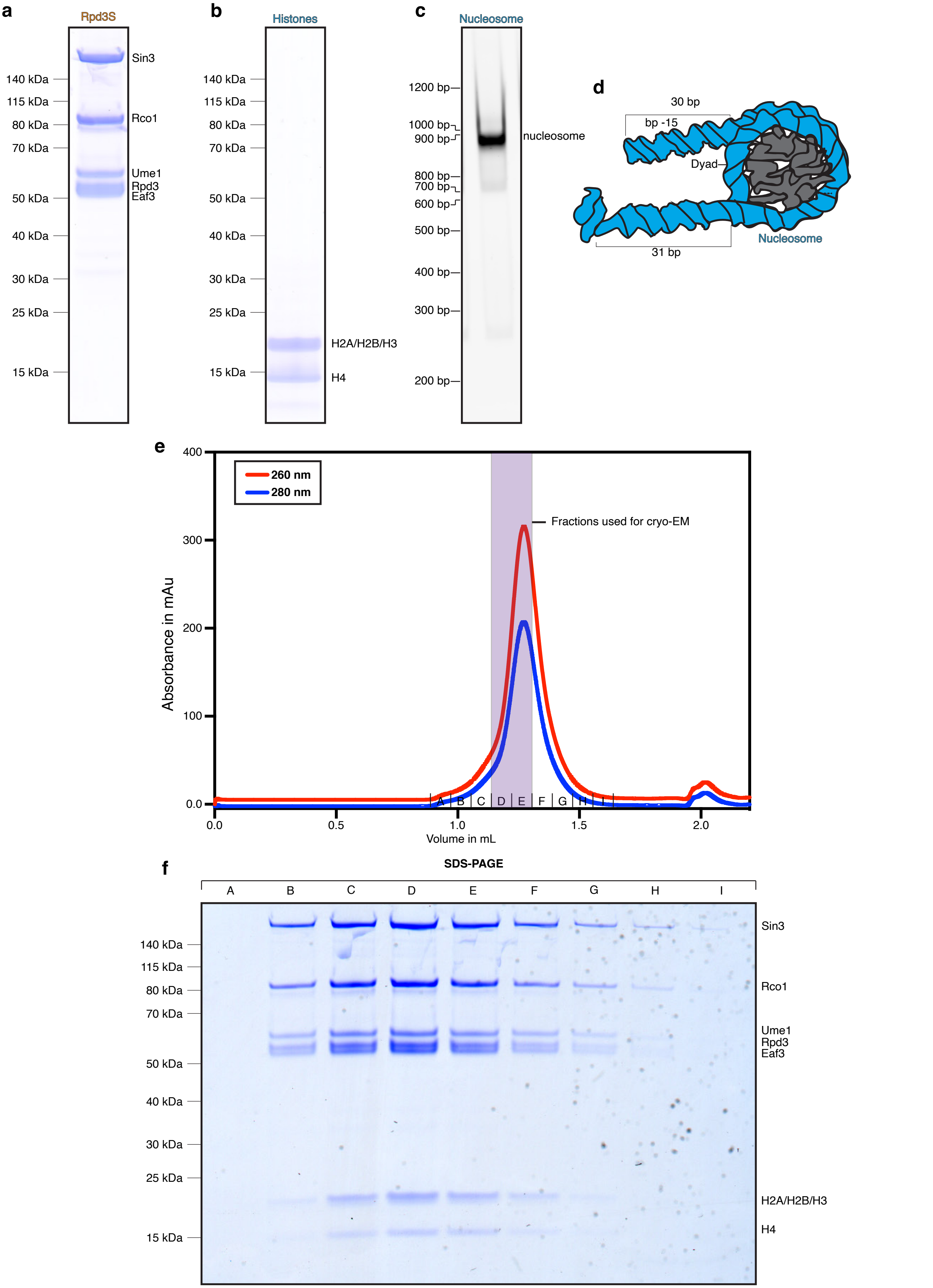


**Supplementary Fig. 1 I Complex formation**

**a,** SDS-PAGE of purified Rpd3S complex. **b,** SDS-PAGE of assembled histone octamer. **c,** Native TBE gel of reconstituted nucleosomal substrate. **d,** Schematic of the employed nucleosomal substrate. Extranucleosomal DNA length is indicated. **e,** Chromatogram of Rpd3S-nucleosome complex formation. Absorbance values at 280 and 260 nm are indicated. A Superose 6 Increase 3.2/300 was used for the complex formation. Fractions used for cryo-EM are indicated. **f,** SDS-PAGE of fractions from complex formation as shown in e. Fractions on gel correspond to indicated fractions in e. Source data are provided as a Source Data file.

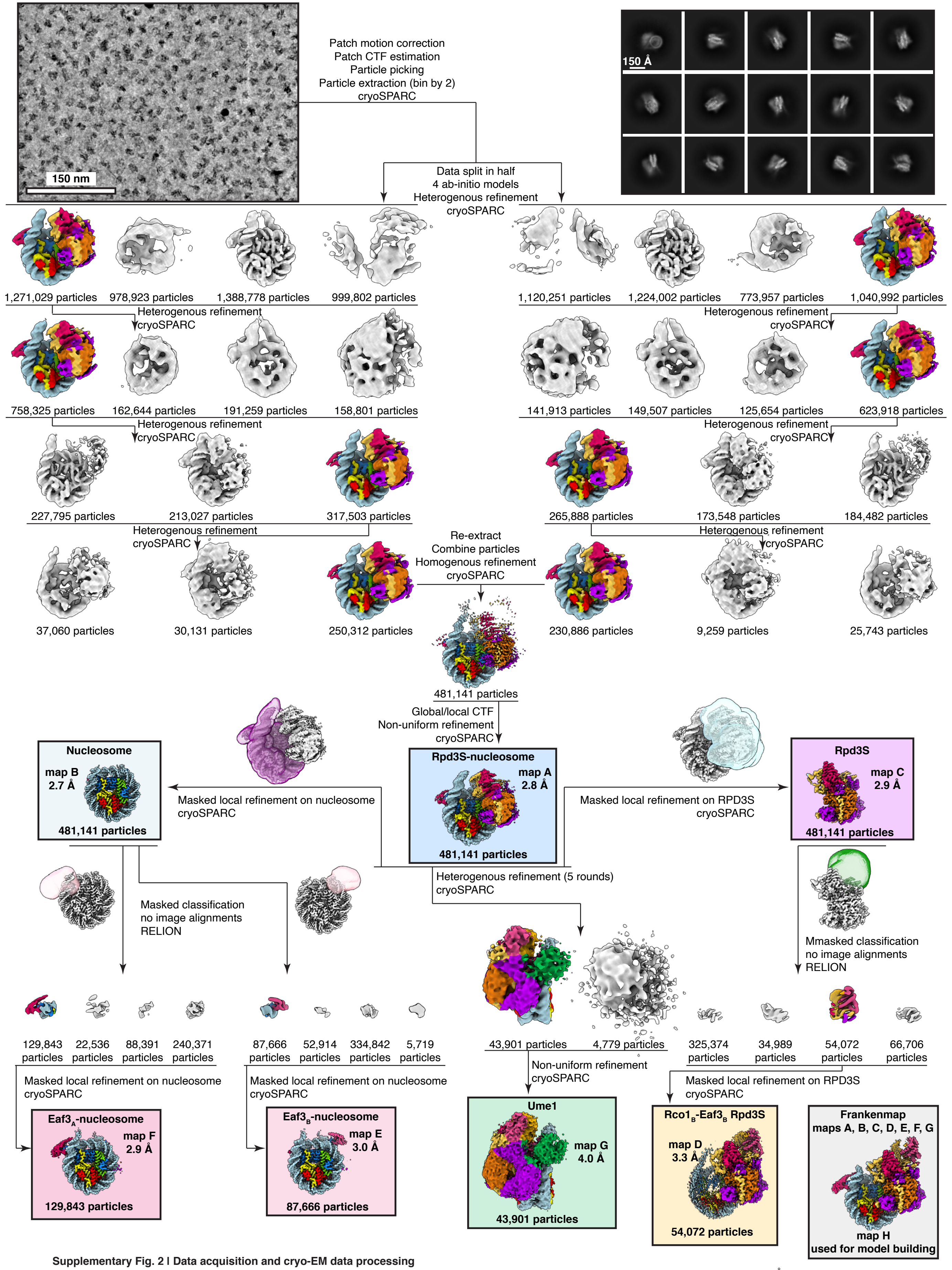


**Supplementary Fig. 2 | Data acquisition and cryo-EM data processing**
Representative micrograph of data collection with scale bar (150 nm), representative 2D classes of Rpd3S-nucleosome complex with scale bar (150 Å), and sorting and classification tree of single-particle cryo-EM data analysis. Employed maps, refinement/classification masks and resolutions are indicated. Map H was used for model building.

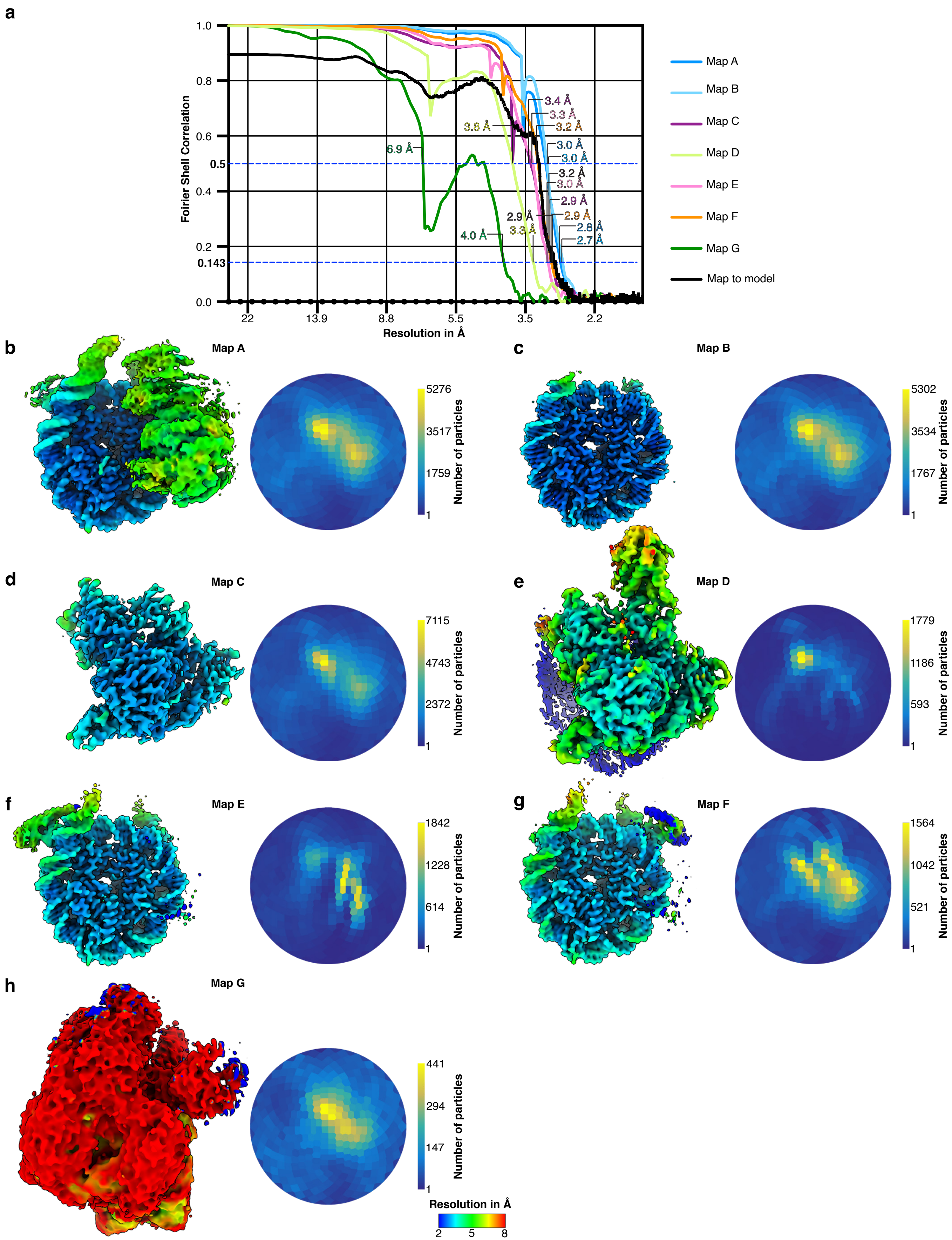


**Supplementary Fig. 3 l FSC curves, angular distribution, and local resolution plots**
**a,** FSC curves of maps A-G. FSC 0.5 and 0.143 criterion are indicated. **b-h,** Local resolution of maps A-G and corresponding angular distribution plots.

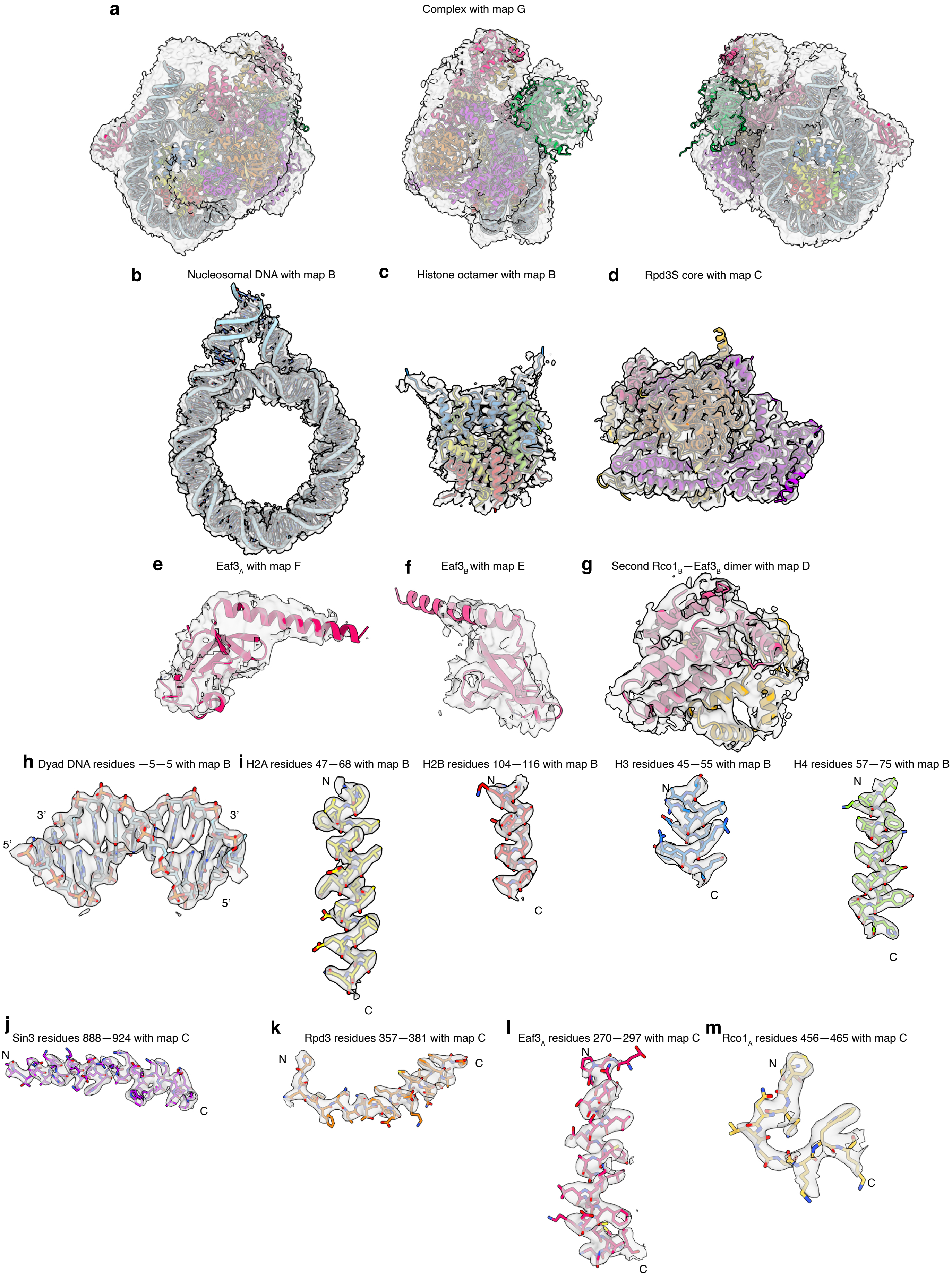


**Supplementary Fig. 4 | Representative densities and density fit**

**a,** Coulomb potential map of Rpd3S-nucleosome complex with atomic model in map G. **b,** Coulomb potential map of nucleosomal and extranucleosomal DNA with atomic model in map B. **c,** Coulomb potential map of histone octamer with atomic model in map B. **d,** Coulomb potential map of Rpd3S core module with atomic model in map C. **e,** Coulomb potential map of Eaf3A chromodomain with atomic model in map F. **f,** Coulomb potential map of Eaf3B chromodomain with atomic model in map E. **g,** Coulomb potential map of Rco1B-Eaf3B (auxiliary module) with atomic model in map D. **h,** Nucleosomal DNA dyad residues with atomic model in map B. **i,** Representative coulomb potential maps of histones H2A, H2B, H3, and H4 with atomic model in map B. **j,** Representative coulomb potential map of Sin3 (residues 888-924) with atomic model in map C. **k,** Representative coulomb potential map of Rpd3 (residues 357-381) with atomic model in map C. **l,** Representative coulomb potential map of Eaf3A (residues 270-297) with atomic model in map C. **m,** Representative coulomb potential map of Rco1A (residues 456-465) with atomic model in map C.

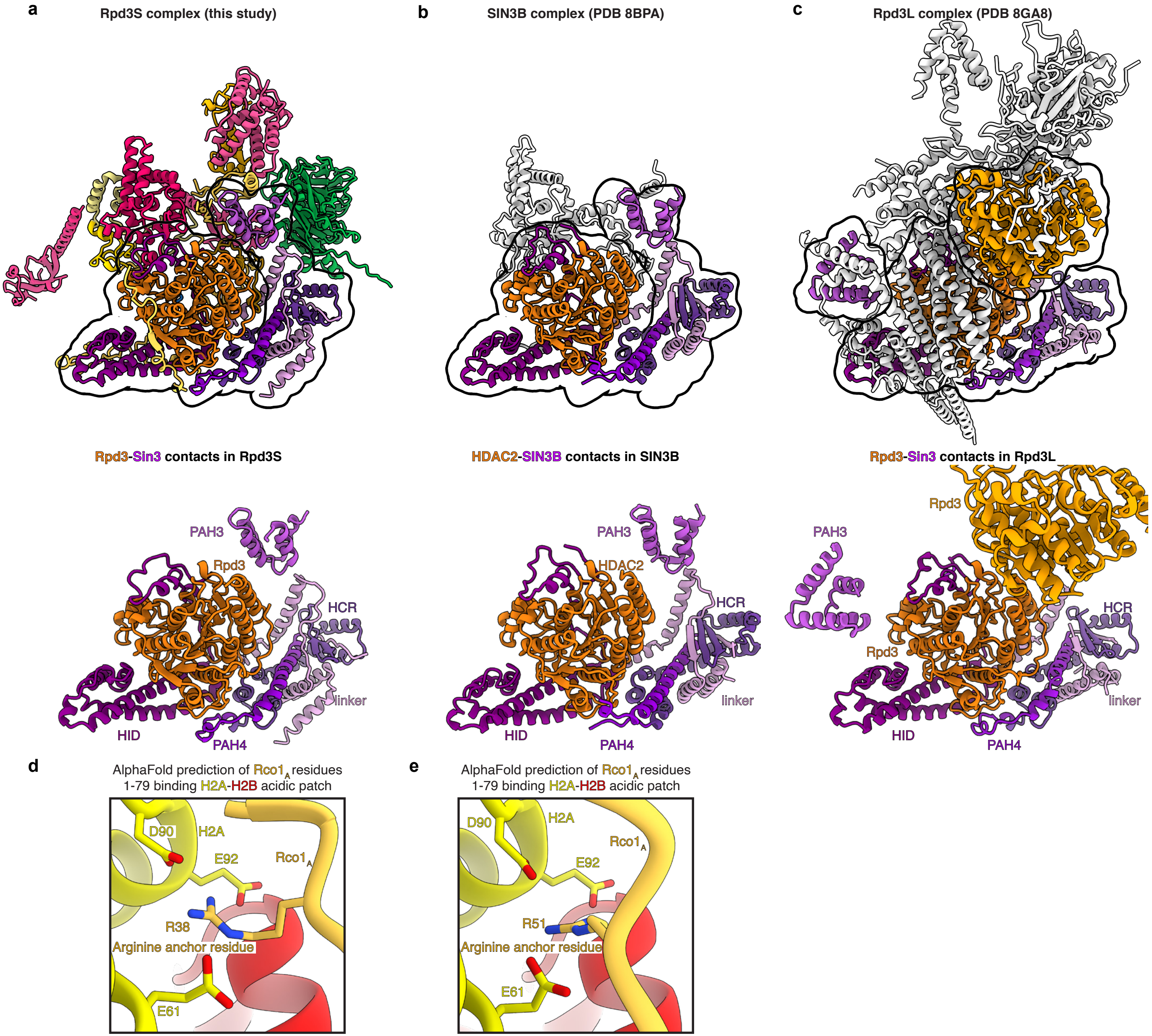


**Supplementary Fig. 5 | Comparison of S. cerevisiae Rpd3S complex with H. sapiens SIN3B and S. cerevisiae Rpd3L complex.**
**a,** S. cerevisiae Rpd3S complex with Rpd3-Sin3 contacts. **b,** H. sapiens SIN3B with HDAC2-SIN3 contacts (PDB code 8BPA). **c,** S. cerevisiae Rpd3L complex with Rpd3-Sin3 contacts (PDB code 8GA8). **d, e** AlphaFold2 multimer predictions of H2A–H2B dimer with Rco1 (residues 1-79) reveal binding of potential arginine anchors into the H2A–H2B acidic patch

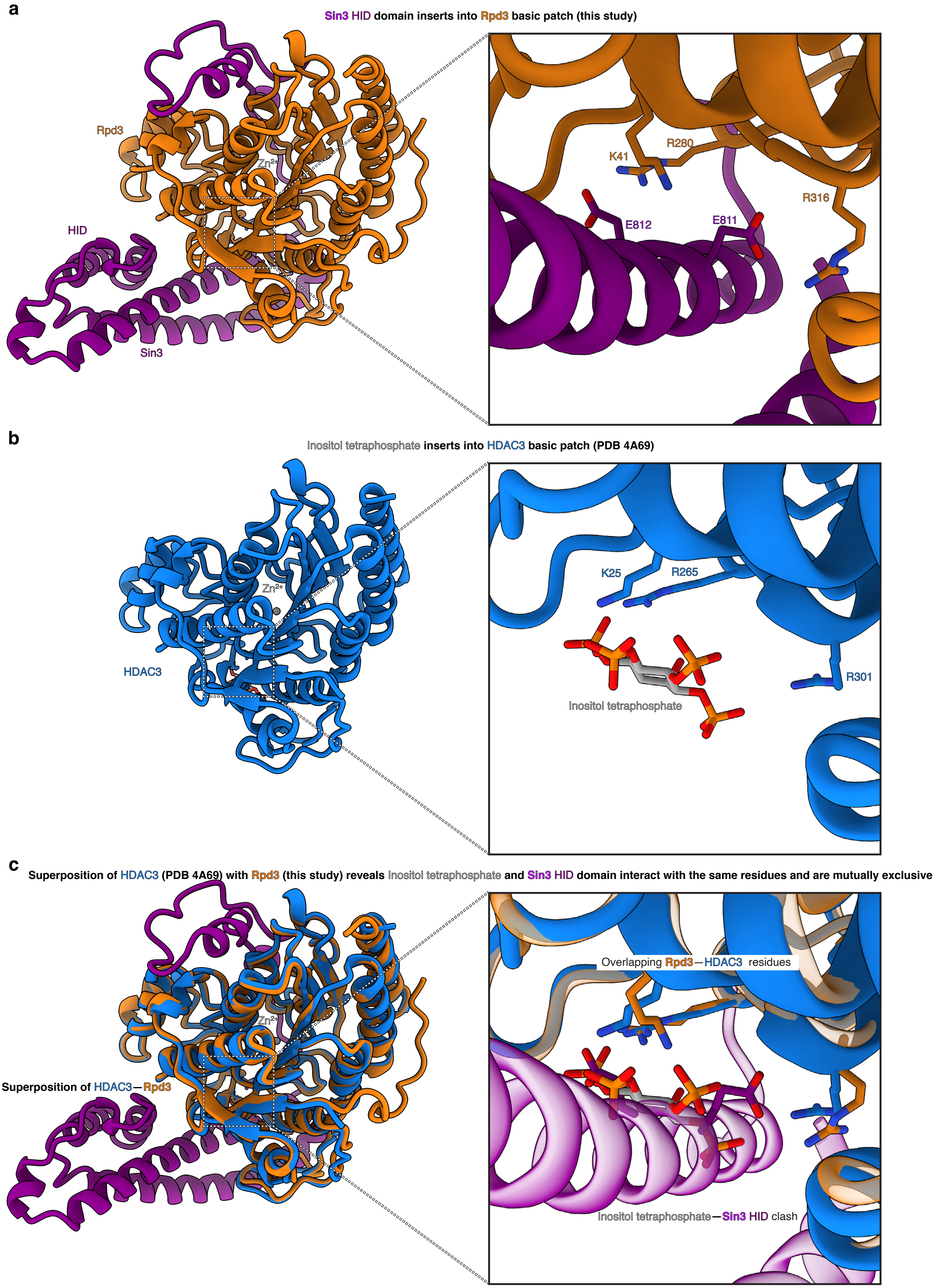


**Supplementary Fig. 6 | Comparison of Rpd3S with HDAC3 unveils Sin3's role in bypassing the need for inositol phosphate in the Rpd3S complex**
**a,** Sin3 binds against Rpd3 in the Rpd3S complex and forms an extensive interaction network. **b,** In HDAC3, inositol tetraphosphate inserts into a pocket formed by Rpd3 (PDB code 4A69) **c,** Overlay of the Rpd3S-nucleosome complex structure reveals that Sin3 occupies the inositol phosphate pocket. Sin3 and inositol phosphate binding to Rpd3 is mutually exclusive.

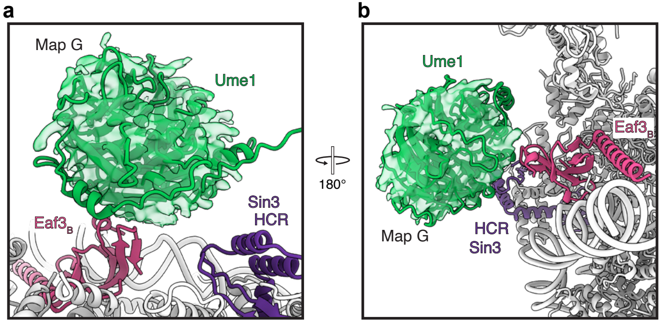


**Supplementary Fig. 7 | The Ume1 subunit is in close proximity to the Eaf3b CHD and the Sin3 HRC domain**. **a-b,** Density (map G, low-pass filtered) and atomic model for Ume1 are shown in green and are located closely to the Sin3 HCR and the Eaf3B chromodomain.

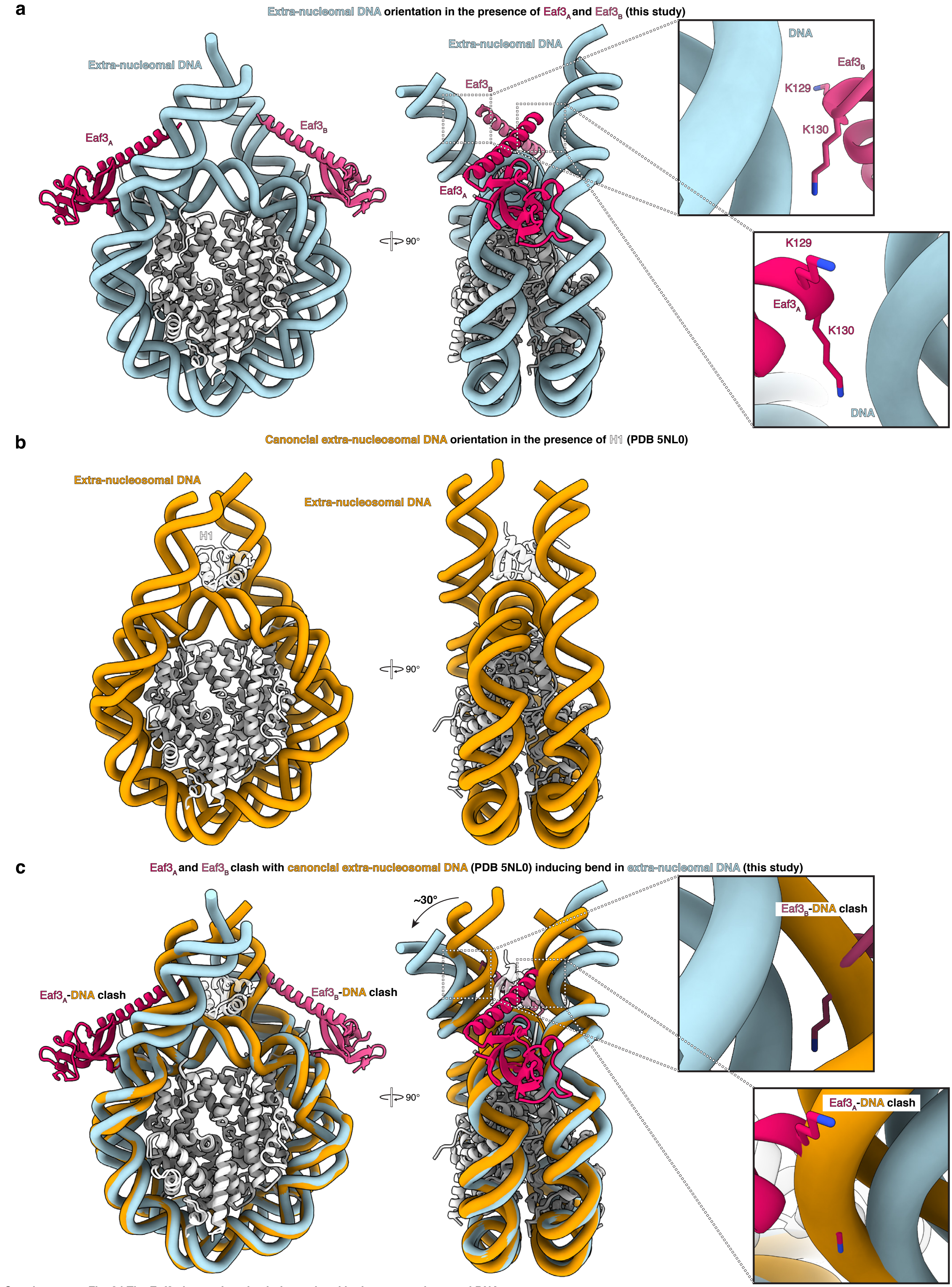


**Supplementary Fig. 8 | The Eaf3 chromodomains induce a bend in the extranucleosomal DNA.**
**a,** The Eaf3 chromodomains interact with extranucleosomal DNA on both sides of the nucleosome. Interactions of Eaf3 with the extranucleosomal DNA is indicated for both Eaf3A and Eaf3B. **b,** Canonical nucleosome in the presence of H1 (PDB code 5NL0)52. **c,** Overlay of canonical nucleosome with Rpd3S-bound nucleosome shows that Eaf3 induces a ~30° bend in the nucleosomal DNA as Eaf3 cannot be accommodated in a canonical nucleosomal DNA conformation in the presence of H1.

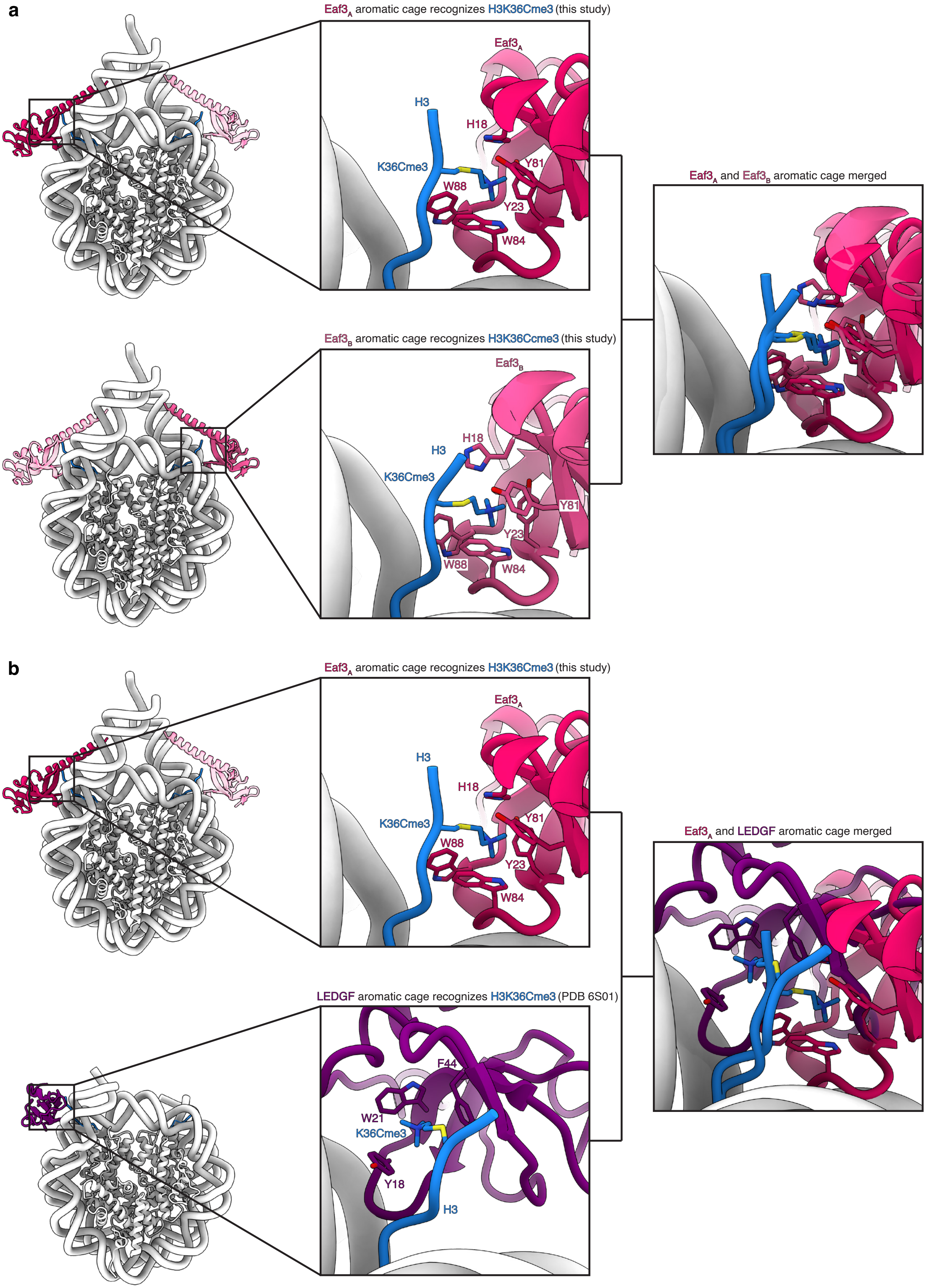


**Supplementary Fig. 9 I Eaf3 binds the H3K36Cme3 analogue.**
**a,** Binding of the EAF3A/B chromodomains to the H3K36Cme3 analogue. **b,** Comparison of the different binding modes of the Eaf3 chromodomains and LEDGF PWWP domain to the H3K36Cme3 analogue. The Eaf3 chromodomains and the LEDGF PWWP domain bind both DNA gyres.

**Supplementary Table 1. Cryo-EM data collection statistics**

| **Data collection and Processing** | | | | |
|---|---|---|---|---|
| Microscope | Thermo Fisher Titan Krios | | | |
| Voltage (keV) | 300 | | | |
| Camera | Gatan K3 | | | |
| Magnification | 105,000 | | | |
| Pixel size at detector (Å per pixel) | 0.822 | | | |
| Total electron exposure ($e^{-}$ Å$^{-2}$) | 51.10 | | | |
| Exposure rate ($e^{-}$ Å$^{-2}$ $s^{-1}$) | 17.983 | | | |
| Number of frames collected during exposure | 50 | | | |
| Defocus range (µm) | 0.5-2.25 | | | |
| Automation software | EPU | | | |
| Energy filter slit width (eV) | 20 | | | |
| Micrographs collected (no.) | 32,564 | | | |
| Micrographs used (no.) | 30,088 | | | |
| Total extracted particles (no.) | 8,797,734 | | | |
| | Rpd3S-nucleosome (Map A) EMD-41449 | Nucleosome (Map B) EMD-41449 | Rpd3S (Map C) EMD-41449 | Rco1$_B$-Eaf3$_B$-Rpd3S (Map D) EMD-41449 |
| Final particles (no.) | 481,141 | 481,141 | 481,141 | 54,072 |
| Resolution (global, Å) | | | | |
| FSC 0.5 masked | 3.0 | 3.0 | 3.4 | 3.8 |
| FSC 0.143 masked | 2.8 | 2.7 | 2.9 | 3.3 |
| Map sharpening *B* factor (Å$^2$) | 82.6 | 74.5 | 84.4 | 62.8 |
| 3DFSC sphericity | 0.967 | 0.970 | 0.875 | 0.866 |
| Map sharpening methods | cryoSPARC | cryoSPARC | cryoSPARC | cryoSPARC |

| | Eaf3$_B$-nucleosome (Map E) EMD-41449 | Eaf3$_A$-nucleosome (Map F) EMD-41449 | Ume1 (Map G) EMD-41449 | Composite map (Map H) EMD-41449 |
|---|---|---|---|---|
| Final particles (no.) | 87,666 | 129,843 | 43,901 | — |
| Resolution (global, Å) | | | | |
| FSC 0.5 masked | 3.3 | 3.2 | 6.9 | — |
| FSC 0.143 masked | 3.0 | 2.9 | 4.0 | — |
| Map sharpening *B* factor (Å$^2$) | 56.4 | 62.7 | 58.2 | 25 |
| 3DFSC sphericity | 0.906 | 0.955 | 0.619 | 0.909 |
| Map sharpening methods | cryoSPARC | cryoSPARC | cryoSPARC | cryoSPARC |

**Supplementary Table 2. Model composition, refinement, and validation.**

| | RPD3S-nucleosome<br>PDB-8TOF<br>EMD-41449 |
|---|---|
| **Model composition** | |
| Non-hydrogen atoms (no.) | 29,102 |
| Protein residues (no.) | 2,697 |
| Ligands (no.) | 7 |
| Nucleotide residues (no.) | 352 |
| | |
| **Model Refinement** | |
| Initial models used (PDB #) | 3LZ0, 8I02 |
| Refinement packages | Coot, PHENIX |
| | |
| Model-Map scores | |
| Cross-correlation coefficient | 0.79 |
| Model resolution (Å) | 2.9 |
| FSC threshold | 0.143 |
| Mean *B* factors ($Å^2$) | Composite map values |
| Protein residues (no.) | 113.48 |
| Ligands (no.) | 180.45 |
| Nucleotide residues (no.) | 81.35 |
| R.m.s. deviations from ideal values | |
| Bond lengths (Å) | 0.010 |
| Bond angles (°) | 1.176 |
| | |
| **Validation** | |
| MolProbity score | 2.40 |
| CaBLAM outliers (%) | 4.14 |
| Clashscore | 11.44 |
| Poor rotamers (%) | 3.09 |
| C-beta deviations (%) | 0.08 |
| EMRinger score | 2.65 |
| Ramachandran plot | |
| Favored (%) | 92.95 |
| Allowed (%) | 6.59 |
| Outliers (%) | 0.45 |

**Supplementary Table 3 | Input structural models and model confidence**

| **Complex/domain** | **Chain id(s)** | **Input model** | **Level of confidence** | **Complex (PDB)** |
|---|---|---|---|---|
| Sin3 | A | *De novo*, Alphafold, 8I02 | Atomic | 8TOF |
| Rpd3 | B | *De novo*, Alphafold, 8I02 | Atomic | 8TOF |
| Ume1 | C | Alphafold, 8I02 | Rigid body dock | — |
| Eaf3A | D | *De novo*, Alphafold, 8I02 | Atomic | 8TOF |
| Eaf3B | E | *De novo*, Alphafold, 8I02 | Secondary | 8TOF |
| Rco1A | G | *De novo*, Alphafold, 8I02 | Atomic | 8TOF |
| Rco1B | F | *De novo*, Alphafold, 8I02 | Secondary | 8TOF |
| Rco1 Acidc patch binding | H | *De novo* | Atomic/clipped | 8TOF |
| H3 | a,e | 3LZ0 | Atomic | 8TOF |
| H4 | b,f | 3LZ0 | Atomic | 8TOF |
| H2A | c,g | 3LZ0 | Atomic | 8TOF |
| H2B | d,h | 3LZ0 | Atomic | 8TOF |
| DNA | N | *De novo*, 3LZ0 | Atomic | 8TOF |
| DNA | T | *De novo*, 3LZ0 | Atomic | 8TOF |
| Active site peptide | x | *De novo* | Atomic/clipped | 8TOF |

Supplementary Figure 1a,b

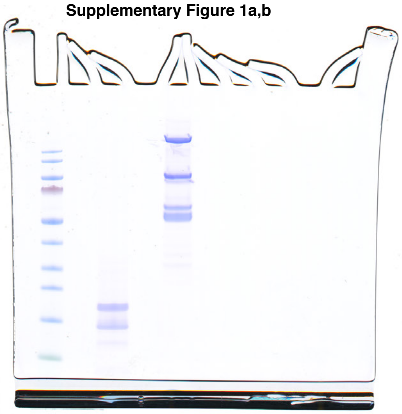

Supplementary Figure 1c

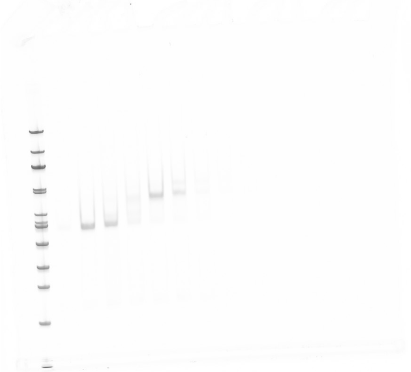

Supplementary Figure 1f

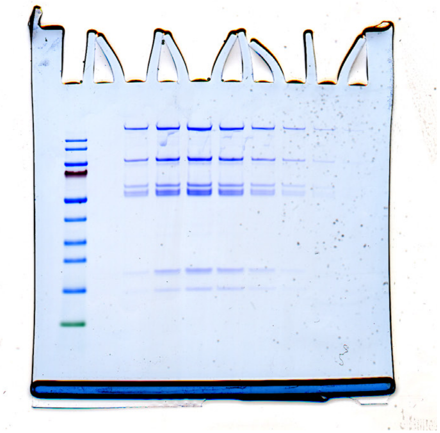

**Source Data Fig. 1 | SDS-PAGE and TBE gels of the Rpd3S complex, histones, and assembled nucleosome**
