# Supplementary material for: Structure of the complete Saccharomyces cerevisiae Rpd3S-nucleosome complex
Source: Nat Commun. 2023 Dec 8;14:8128. doi: 10.1038/s41467-023-43968-8 (PMC10709384; doi:10.1038/s41467-023-43968-8)
Supplement: Supplementary file 3 — Description of Additional Supplementary Files [file 41467_2023_43968_MOESM3_ESM.docx]

**Supplementary Movie 1**

Cryo-EM density and model of Rpd3S-nucleosome complex, The Cryo-EM density and corresponding model of the Rpd3S complex is presented with subunits colored accordingly.
